# Influence of Perinatal Factors on Gene Expression of IAPs Family and Main Factors of Pluripotency: *OCT4* and *SOX2* in Human Breast Milk Stem Cells—A Preliminary Report

**DOI:** 10.3390/ijms24032476

**Published:** 2023-01-27

**Authors:** Paulina Gil-Kulik, Michał Leśniewski, Karolina Bieńko, Monika Wójcik, Marta Więckowska, Dominika Przywara, Alicja Petniak, Adrianna Kondracka, Małgorzata Świstowska, Rafał Szymanowski, Agnieszka Wilińska, Mateusz Wiliński, Bartosz J. Płachno, Marzena Kostuch, Mansur Rahnama-Hezavach, Mariusz Szuta, Anna Kwaśniewska, Anna Bogucka-Kocka, Janusz Kocki

**Affiliations:** 1 Department of Clinical Genetics, Medical University of Lublin, 11 Radziwillowska Str., 20-080 Lublin, Poland; 2 Student Scientific Society of Clinical Genetics, Medical University of Lublin, 11 Radziwillowska Str., 20-080 Lublin, Poland; 3 Department of Obstetrics and Pathology of Pregnancy, Medical University of Lublin, 11 Staszica Str., 20-081 Lublin, Poland; 4 Department of Plant Cytology and Embryology, Institute of Botany, Faculty of Biology, Jagiellonian University in Kraków, 9 Gronostajowa St., 30-387 Cracow, Poland; 5 Department of Neonatology, Independent Public Clinical Hospital No. 4, 8 Jaczewskiego St., 20-954 Lublin, Poland; 6 Chair and Department of Dental Surgery, Medical University of Lublin, 6 Chodzki St., 20-093 Lublin, Poland; 7 Chair of Oral Surgery, Jagiellonian University Medical College, 4 Montelupich St., 31-155 Kraków, Poland; 8 Chair and Department of Biology and Genetics, Medical University of Lublin, 4a Chodźki St., 20–093 Lublin, Poland

**Keywords:** breast milk stem cells, IAPs, BIRC, OCT4, SOX2, gene expression

## Abstract

Due to their therapeutic potential, mesenchymal stem cells are the subject of intensive research on the use of their potential in the treatment of, among others, neurodegenerative diseases or immunological diseases. They are among the newest in the field of medicine. The presented study aimed to evaluate the expression of eight genes from the IAP family and the gene regulating IAP—*XAF1*—in stem cells derived from human milk, using the qPCR method. The relationships between the expression of genes under study and clinical data, such as maternal age, maternal BMI, week of pregnancy in which the delivery took place, bodyweight of the newborn, the number of pregnancies and deliveries, and the time elapsed since delivery, were also analyzed. The research was carried out on samples of human milk collected from 42 patients hospitalized in The Clinic of Obstetrics and Perinatology of the Independent Public Clinical Hospital No. 4, in Lublin. The conducted research confirmed the expression of the following genes in the tested material: *NAIP, BIRC2, BIRC3, BIRC5, BIRC6, BIRC8, XIAP, XAF1, OCT4* and *SOX2*. Moreover, several dependencies of the expression of individual genes on the maternal BMI (*BIRC5, XAF1* and *NAIP*), the time since childbirth (*BIRC5, BIRC6, XAF1* and *NAIP*), the number of pregnancies and deliveries (*BIRC2, BIRC5, BIRC6* and *XAF1*), the manner of delivery (*XAF1* and *OCT4*), preterm labor (*BIRC6* and *NAIP*) were demonstrated. Additionally, we found positive relationships between gene expression of BIRC7, BIRC8 and XAF1 and the main factors of pluripotency: *SOX2* and *OCT4*. This work is the first to investigate the expression of genes from the IAPs family in mother’s milk stem cells.

## 1. Introduction

Stem cells belong to the group of non-specialized cells. They show the ability to self-renew. They can also differentiate into any cell of the human body, and this ability diminishes as the degree of specialization increases. The population of human breast milk stem cells was first described in 2007 and defined as hBSC (human breast milk stem cells) [1,2,3,4].

The role of milk stem cells has not yet been fully explained [2]. Therefore, it is very important to learn about the immune, proliferative and viability of milk stem cells in order to be able to fully use their regenerative potential in regenerative medicine and, thanks to their anti-inflammatory properties, in the treatment of autoimmune diseases. hBr-MSC are potently able to differentiate into the mesoderm, ectoderm, and endoderm cell lineages [5].

A characteristic feature of stem cells is the high expression of the main pluripotency factors such as OCT4 or SOX2. OCT4, in close collaboration with SOX2 and NANOG, have an important regulatory function in the transcription and maintenance of stem cell pluripotency by activating the expression of genes related to pluripotency and repressing the expression of genes related to differentiation. They are also key transcription factors necessary for MSC self-renewal and survival. POU5F1 and SOX2 are also the most important factors at the transcription level when reprogramming a human somatic cell to iPS cells [6,7].

Inhibitors of Apoptosis Proteins—IAP—affect the apoptosis process but also the immune and inflammatory response as well as cell mobility, proliferation, and differentiation. They disrupt the transmission of the apoptotic signal in the mechanism of binding with other proteins involved in apoptosis [8,9,10]. Eight IAP proteins have been recognized: XIAP (Human X Chromosome-Encoded IAP), IAP-1/BIRC2 (baculoviral IAP repeat-containing 2), IAP-2/BIRC3, Liwin/BIRC7/ML-IAP (Melanoma IAP), BIRC8/ILP -2 (IAP-like Protein 2), NAIP (Neuronal Apoptosis-Inhibitory Protein), BRUCE/Apollon/BIRC6, and surviving/BIRC5 [10,11].

From the family of eight proteins, XIAP is the best recognized, thanks to the presence of three BIR domains; it shows the strongest anti-apoptotic effect, and it binds and inhibits caspases -3, -7, and -9 [12]. Most of the IAP family proteins influence cell signaling. It has been shown that cIAP1, cIAP2, and XIAP regulate the innate immune response. [13].

The cIAP1 protein, through the BIR1 domain, connects with TNF-receptor-associated factor 2 (TRAF2), which is required in several signaling pathways. The cIAP1 and cIAP2 proteins contain the caspase recruitment domain (CARD), which influences the interactions between proteins. CIAP1 protects progenitor cells from TRAIL-induced apoptosis by inhibiting caspase 3 activation. Presumably through the RING domain, cIAP1 and cIAP2 act as E3 ubiquitin ligases for nuclear factor inducing kinase (NF-κB). As a result, they participate in the expression of survival-defining molecules and take part in the secretion of fibronectin [14,15,16]. IAP E3 ligase activity also affects the regulation of cell shape, migration, activity, and differentiation [17]. Studies show that cIAP1 and cIAP2 affect signaling associated with innate immunity. Their deficiency reduces caspase 1 activation and suppresses the inflammatory response in response to inflammasome agonists [14,18]. Inflammasomes recognize infection and initiate inflammation through caspase-1 activation [19]. The immune response is based on the activity of ubiquitin ligase and the regulation of NF-κB, MAPK, TNFR (tumor necrosis factor receptor), IRF. Ubiquitination is a post-translational modification of proteins. It plays an important role in the process of apoptosis and signal transduction [20].

Survivin contains only one functional domain—BIR. BIRC5, apart from the inhibition of apoptosis, takes part in the regulation of the cell cycle. This is evidenced by the loss of protein causing mitotic arrest or catastrophe in cells. Apollon’s mechanism of action is not yet fully understood. In the literature, we can find that Apollon’s deficiency in mice leads to embryonic death due to placental apoptosis [15,21].

X-linked IAP (XIAP) is a potent caspase inhibitor. It binds directly to caspase-9 through the C-terminal domain of the BIR (BIR3), while caspase-3 and caspase-7 bind through BIR2. Research shows that the anti-apoptotic action of XIAP also requires the presence of the C-terminal RING domain. The action of XIAP is antagonized by mitochondrial proteins. XIAP overexpression occurs in neoplastic diseases and influences the survival of neoplastic tumors [15].

No information has been found in the literature on the study of the expression of genes from the IAPs family in human stem cells derived from breast milk, the presented paper is the first report.

IAP apoptosis inhibitors are well researched in neoplastic diseases; their high expression in cancer cells causes disturbances in the apoptosis process and cell cycle disorders, which is usually associated with a worse response of tumors to the treatment [22].

The study of IAP expression in milk stem cells is a new topic, not previously considered. The physiological role of IAP in stem cells is unknown. In our previous work [14,23], we assessed IAP, among others, in stem cells of the umbilical cord, and there we demonstrated the involvement of the *BIRC5* gene from the IAP family in the physiological processes of stem cells, such as: cell proliferation, cell cycle progression, cell differentiation, survival and maintenance of stem cells.

The study aims to evaluate the expression of eight genes from the IAP family and the gene regulating IAP—*XAF1*—in human stem cells derived from breast milk. The study also assessed the influence of the mother’s age, mother’s BMI, week of pregnancy in which the child was born, bodyweight of the newborn, number (order) of pregnancies and deliveries, and the time elapsed after birth on the expression level of *NAIP, BIRC2, BIRC3, BIRC5, BIRC6, BIRC7, BIRC8, XIAP*, *XAF1, OCT4* and *SOX2* genes, in breast milk stem cells.

## 2. Results

### 2.1. Phenotype Breast Milk Stem Cells

**Figure 1:**
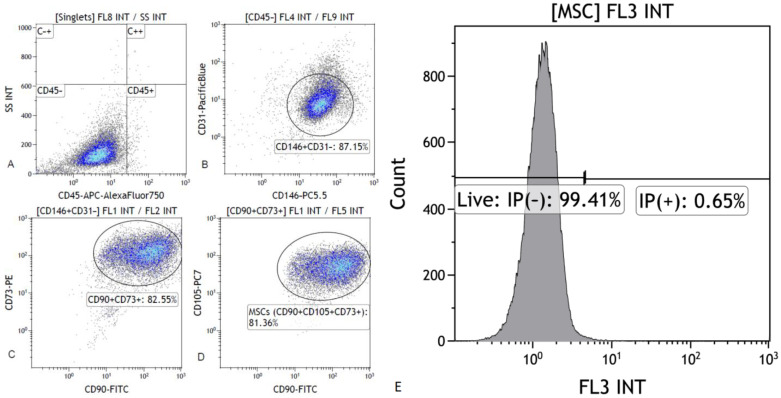
Exemplary analysis of the immunophenotype of milk mesenchymal stem cells after in vitro culture. Cytogram (**A**). Cell population negative for CD45 antigen (“CD45-” Gate). Cytogram (**B**). Percentage of cells positive for CD146 and negative for CD31 (Gate “CD146 + CD31–”). Cytogram (**C**). Percentage of cells expressing CD90 and CD73 (Gate “CD90 + CD73 +). Cytogram (**D**). Percentage of milk mesenchymal stem cells positive for CD90, CD105, CD73 (Gate “CD90 + CD105 + CD73 +). (**E**). Cytometric evaluation of the viability of stem cells after cell culture, using propidium iodide.

**Figure 2:**
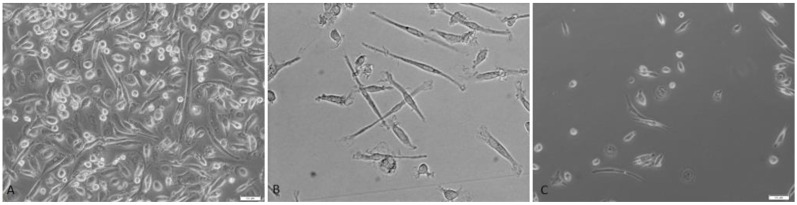
(**A**) Heterogeneous late milk cell population at the beginning of cell culture (20× magnification). (**B**) Colostrum mesenchymal stem cell population during cell culture (100× magnification). (**C**) Mesenchymal stem cell population during 48 h cell culture (20× magnification). Bright field microscopy (Xcellence RT system with an IX81 inverted microscope Olympus).

The immunophenotype (CD73, CD90, CD105, and CD146 expression) and cell viability of milk mesenchymal stem cells was confirmed cytometrically (Figure 1). The fibroblast-like shape of the MSC from mothers milk and the adherence to the plastic walls were confirmed (Figure 2).

### 2.2. Molecular Analysis Breast Milk Stem Cells

Based on the analysis, it was confirmed that the examined human milk stem cells express, at the transcript level, the following genes: *NAIP, BIRC2, BIRC3, BIRC5, BIRC6, BIRC8, XIAP, XAF1, OCT4* and *SOX2*. Expression of the *BIRC7* gene was obtained only in 13 out of 42 tested trials.

We conducted the analysis of the dependence of the expression of the studied genes in stem cells on clinical parameters such as age and BMI of women from whom milk was collected, number of pregnancies and deliveries, the occurrence of miscarriages, pregnancy week in which delivery took place, route of delivery, the time elapsed from delivery to sampling, sex of the child, bodyweight of the newborn at birth.

#### 2.2.1. Analysis of the Dependence of IAPs and *XAF1* Gene Expression in Breast Milk Stem Cells on Women’s BMI

**Figure 3:**
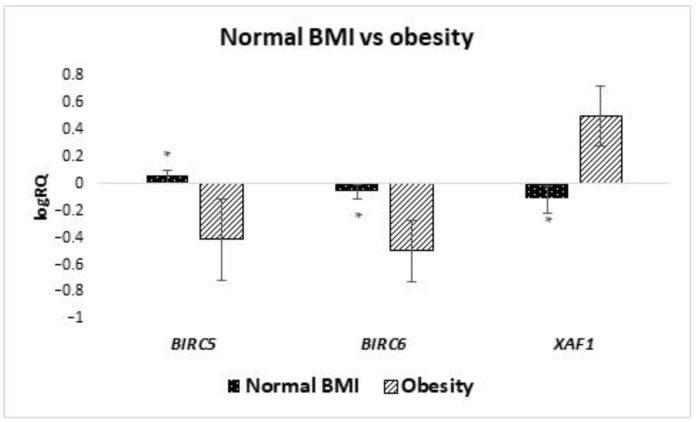
Differences in expression of genes (logRQ ± SE) tested in milk stem cells depending on BMI. * *p* < 0.05 Student’s *t*-test.

The studied group of women was divided into subgroups depending on the size of BMI. It has been shown that the average expression level of *BIRC6* (*p* = 0.028) and *XAF1* (*p* = 0.012) genes in stem cells from the milk of obese women with BMI higher than 30 is statistically significantly higher than in women with normal BMI (Figure 3). On the other hand, when analyzing the mean expression of the *BIRC5* gene, it was observed that it was higher in women with a normal BMI (*p*-value close to statistical significance, *p* = 0.052). In the case of the expression of the *BIRC7* gene, no statistical analysis was performed, due to the insufficient amount of data. Taking into account the other IAP genes, no significant differences were found depending on BMI.

Performing the correlation analysis in milk stem cells showed a strong negative correlation between the level of *BIRC6* gene expression and the BMI value (r = −0.587, *p* < 0.05): the higher the mother’s BMI, the lower the *BIRC6* gene expression observed (Table 1).

#### 2.2.2. Analysis of the Dependence of IAPs and *XAF1* Gene Expression in Milk Stem Cells from the Time of Collection (Time since Delivery)

**Figure 4:**
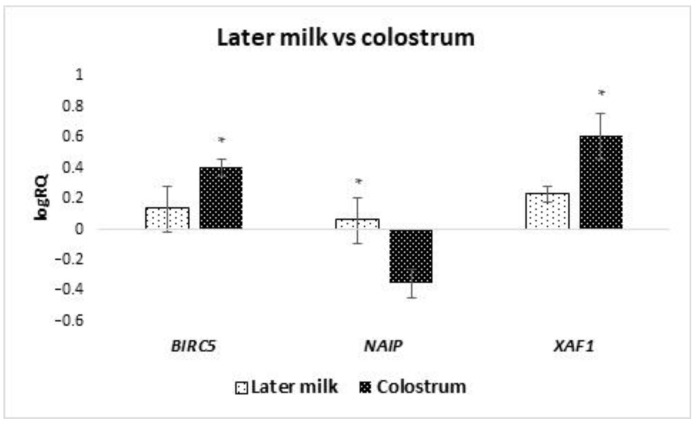
Differences in the expression of genes (logRQ ± SE) tested in milk stem cells by groups of colostrum and milk. * *p* < 0.05 Student’s *t*-test.

**Figure 5:**
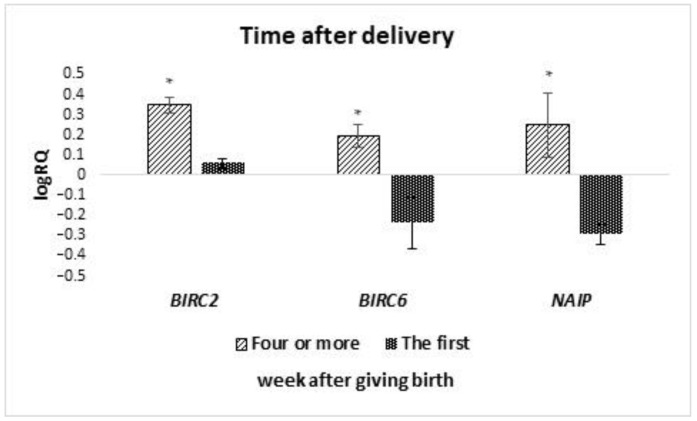
Differences in the expression of genes (logRQ ± SE) tested in milk stem cells depending on the time after delivery. * *p* < 0.05 Student’s *t*-test.

The analyzed groups of cells were divided into subgroups depending on the time of collection for colostrum (collection from 2 to 5 days after delivery) and milk (collection took place after 7 days after delivery). It was noted that the average expression level of the *BIRC5* (*p* = 0.036) and *XAF1* (*p* = 0.029) gene is statistically significantly higher in colostrum compared to the late milk (Figure 4). On the other hand, the average level of *NAIP* gene expression (*p* = 0.025) was statistically significantly lower in colostrum compared to milk. A comparison was also made of the expression level of genes tested in cells isolated from milk collected in the first week after delivery with milk collected in the fourth week or later. It was observed that the mean expression level of the *BIRC2* (*p* = 0.037), *BIRC6* (*p* = 0.014) and, *NAIP* (*p* = 0.020) genes in the stem cells of milk collected in the fourth week after delivery or later is statistically significantly higher compared to the first week (Figure 5). The remaining genes from the IAP family showed no significant differences in the level of expression depending on the time of collection.

The analysis of the search for correlation showed a statistically significant negative correlation between the expression of the *XAF1* gene and the sampling time (the number of days after delivery) (r = –0.494, *p* < 0.05). The more days elapsed after delivery, the lower the *XAF1* expression observed in the milk stem cells (Table 1).

#### 2.2.3. Analysis of the Dependence of IAPs and *XAF1* Gene Expression in Milk Stem Cells on the Number (Sequence) of Pregnancies and Deliveries

**Figure 6:**
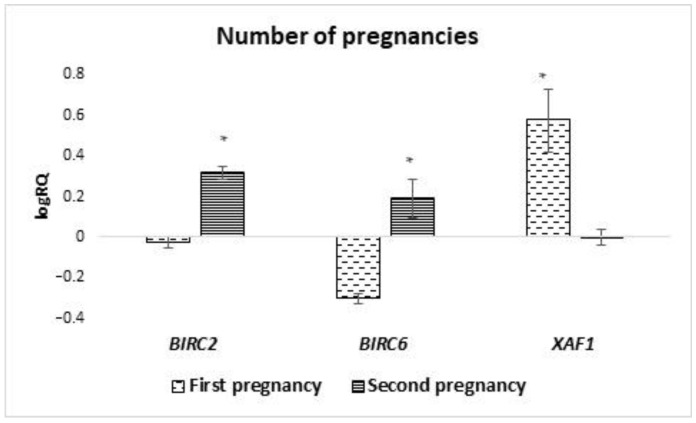
Differences in the expression of genes (logRQ ± SE) tested in milk stem cells depending on the number/sequence of pregnancies. * *p* < 0.05 Student’s *t*-test.

The average level of expression of genes tested in milk stem cells in women for whom it was the first pregnancy/childbirth was compared with women who were pregnant or gave birth for the second time. A statistically significant difference was observed in the mean expression level of the *BIRC2* (*p* = 0.025), *BIRC6* (*p* = 0.011), and *XAF1* (*p* = 0.025) genes. The *BIRC2* and *BIRC6* genes in milk stem cells of women in the second pregnancy were characterized by a higher level of expression compared to stem cells from women who were pregnant for the first time. In the case of *XAF1* expression, an inverse relationship was observed in women who were pregnant for the first time, and the average level of expression of this gene was higher (Figure 6).

**Figure 7:**
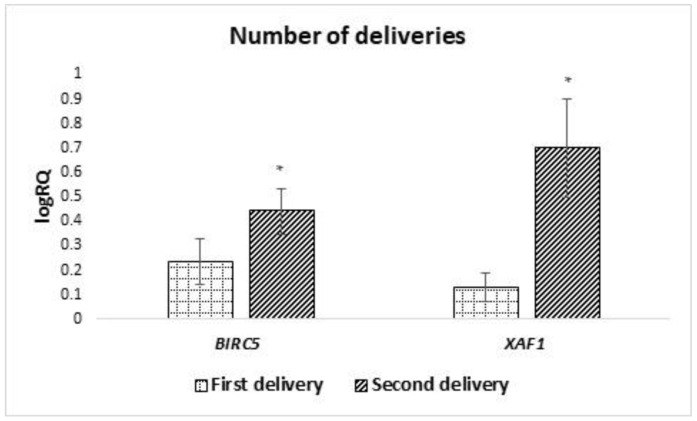
Differences in the expression of genes (logRQ ± SE) tested in milk stem cells depending on the number/order of births. * *p* < 0.05 Student’s *t*-test.

Analyzing the dependence of the expression of the genes under study on the number of deliveries, it was shown that the average level of expression of the *BIRC5* (*p* = 0.040) and *XAF1* (*p* = 0.027) genes in milk stem cells is statistically significantly higher in women giving birth for the second time (Figure 7). The expression of the remaining genes under study did not show any significant dependence on the number of pregnancies and deliveries.

#### 2.2.4. Analysis of the Relationship between the Expression of IAPs and *XAF1* Genes in Milk Stem Cells on the Occurrence of Miscarriages in Women

**Figure 8:**
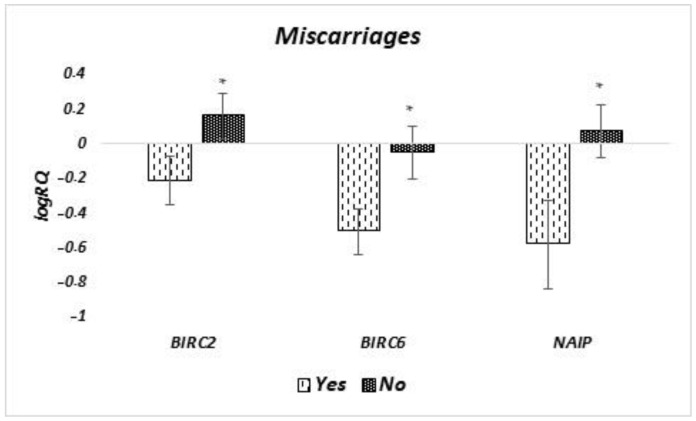
Differences in the expression of genes (logRQ ± SE) tested in milk stem cells depending on the occurrence of miscarriages in women. * *p* < 0.05 Student’s *t*-test.

The analysis of differences in the level of expression of genes tested in milk stem cells, depending on whether the women had had previous miscarriages, showed that the expression of *BIRC2* (*p* = 0.038), *BIRC6* (*p* = 0.045), and *NAIP* (*p* = 0.033) genes is statistically significantly lower in women who experienced miscarriages (Figure 8). In the case of the other genes tested, no significant differences were found.

#### 2.2.5. Analysis of the Dependence of IAPs and *XAF1* Gene Expression in Milk Stem Cells on the Way of Delivery

**Figure 9:**
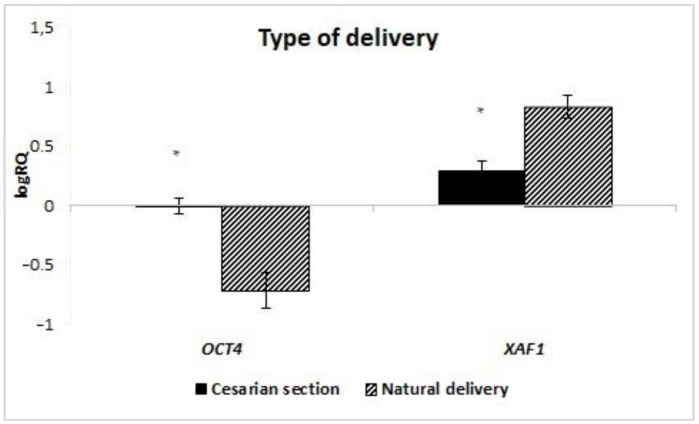
Mean expression of the *XAF1* and *OCT4* (logRQ ± SE) genes in breast milk stem cells depending on the type of delivery. * *p* < 0.05 Student’s *t*-test.

The analysis of the dependence of the expression of genes on the route of delivery showed that in the stem cells of milk obtained from women giving birth by natural force (ND), the average level of *XAF1* (*p* = 0.047) gene expression is statistically significantly higher than in women giving birth by caesarean section. On the other hand, the expression of the *OCT4* gene was statistically significantly higher in stem cells obtained from patients giving birth by caesarean section (CC) (*p* = 0.018) (Figure 9). Taking into account the *BIRC7* gene, no statistical analysis was performed due to insufficient data. The expression of the remaining genes under study did not depend on the manner of delivery.

#### 2.2.6. Analysis of the Dependence of IAPs and *XAF1* Gene Expression in Milk Stem Cells from the Time of Delivery

**Figure 10:**
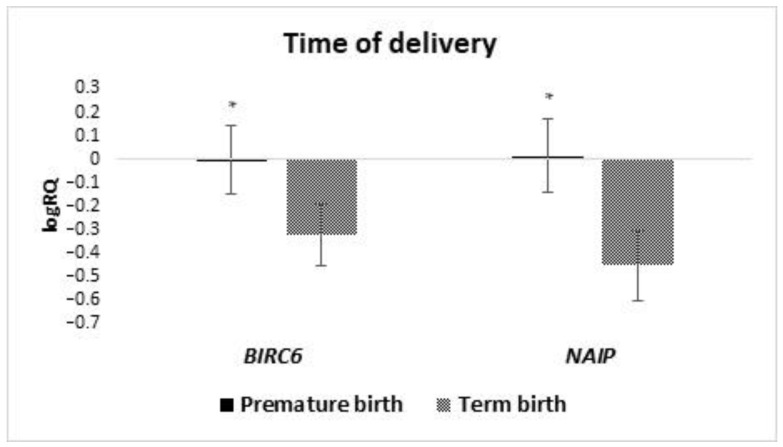
Differences in the tested genes expression (logRQ ± SE) of tested in milk stem cells depending on the prevalence of prematurity. * *p* < 0.05.

An analysis of the expression level of the tested genes depending on the time of childbirth was performed. The group was divided into two subgroups of children born before 37 weeks gestation and those born after 37 weeks gestation. The studies showed that the average expression of *BIRC6* (*p* = 0.049) and *NAIP* (*p* = 0.018) genes in the stem cells of milk from women who gave birth prematurely is statistically significantly higher than in women who gave birth on time (Figure 10). The other tested genes showed no statistically significant differences depending on the time of delivery.

The analysis of correlation showed the existence of positive significant, moderate and strong relationships between the expression levels of *BIRC2* and *BIRC3* (r = 0.449), *BIRC2* and *BIRC6* (r = 0.819), *BIRC2* and *NAIP* (r = 0.650) and *BIRC2* and *XIAP* (r = 0.563), *BIRC3* and *BIRC8* (r = 0.362), *BIRC3* and *NAIP* (r = 0.426), *BIRC3* and *XAF1* (r = 0.488), *BIRC5* and *BIRC6* (r = 0.396), *BIRC5* and *BIRC7* (r = 0.648), *BIRC6* and *NAIP* (r = 0.577), *BIRC6* and *XIAP* (r = 0.543), *BIRC8* and *XAF1* (r = 0.484), *OCT4* and *BIRC7* (r = 0.591), *OCT4* and *BIRC8* (r = 0.821), *OCT4* and *XAF1* (r = 0.568), *OCT4* and *SOX2* (r = 0.962), *SOX2* and *BIRC8* (r = 0.798), *XAF1* and *SOX2* (r = 0.492). Moreover, a statistically significant positive correlation between the expression level of the *XAF1* gene and maternal age (r = 0.438) was observed; it was observed that the higher the maternal age, the higher the expression of the *XAF1* gene in milk stem cells (Table 1).

In the course of the conducted research, no influence of the bodyweight and sex of the newborn on the level of expression of the studied genes was found.

## 3. Discussion

The specific ingredients of milk meet the nutritional needs of the newborn. While the beneficial effects of breast milk on a child’s basic physical and intellectual development in the short and long term have been known for centuries, the mechanism remains uncertain. The mechanism by which breast milk consumed early in life provides protection against diseases that occur later in life needs to be investigated. The composition of breast milk varies among women. Lactation stage, degree of satiety, breastfeeding and pumping, the infant’s growth rate and needs, the health of the mother and the environment cause differences in the composition of breast milk [24,25].

Milk regulates the immunity of newborns and infants through natural bioactive and immunological ingredients and provides protection against infections [26]. Recent studies have shown that the components of human milk establish communication with other cells during the development and regulation of the innate and acquired immune systems [27].

Stem cells in breast milk were first described in 2007. Cregan et al. [4] reported the presence in breast milk of general marker of nestin stem cells, which is a marker of nerve, myeloid, pancreatic and epithelial stem cells.

Our research confirms the presence of stem cells in breast milk. The analysis of the expression levels of the IAP and *XAF1* family genes in the tested material shows that there is a correlation between the expression levels of individual genes and the clinical data. The genes of the IAP family have never before been studied in human milk stem cells. According to literature data, the number of stem cells in breast milk may differ depending on the gestational age. The changes taking place in the composition of breast milk are to support the child’s development in the best possible way [28].

In a study by Briere et al. [29], it was shown that the values of stem cell markers did not differ significantly between the groups, but the stem cell markers SOX2, Nanog, CD90 and CD105 were more expressed in milk of premature infants than in term milk. Although the cause is not fully elucidated, it is assumed that the symbiotic relationship between mother and child continues during breastfeeding, and there are changes in stem cells and gene expression levels depending on the infants needs [30].

The role of IAP in mesenchymal stem cells is not clear. Our previous studies suggest that BIRC5 may be responsible for the pluripotency state of stem cells, and its high expression may also be responsible for the de-differentiation of cancer cells [23]. In our previous works, we suggest that the method of delivery and the biophysical parameters of umbilical cord blood, as well as the age of the woman giving birth, significantly affect the expression of genes from the IAP family and, thus, the clinical usefulness of the obtained cells. We have previously shown that in younger women who give birth naturally and in the acidic environment of umbilical cord blood, MSCs are characterized by higher expression of *BIRC2, BIRC3* and *BIRC5* genes [14]. Considering the possible functions of the proteins encoded by the studied genes, we speculate that mesenchymal stem cells collected from Wharton’s umbilical cord jelly from younger women giving birth naturally probably have greater clinical utility and higher therapeutic potential due to their more maternal nature, greater potential for differentiation, greater ability to adhere and migrate, and greater resistance to apoptosis.

Knowledge about the role of IAP in somatic stem cells is extremely important, not only because of the impact of IAP levels on the viability, proliferative or differentiation potential of stem cells, but also in the context of cancer therapy aimed at inhibiting IAP expression. The demonstrated possible involvement of IAP in maintaining the pluripotency of somatic stem cells will revolutionize the approach to cancer treatment using IAP inhibitors and will help to better predict the side effects of the therapy used. The elucidation of the physiological mechanisms of IAP regulation in normal stem cells will also help to better understand the pathomechanism of cancer and, thus, improve therapeutic options [31]. *BIRC7* is also part of the IAP, but it also has another feature. Due to the strong stimulation of the cell to apoptosis, *BIRC7* is cleaved by caspase 3 and 7, thus creating a truncated form—Asp52—having opposite properties, i.e., anti-apoptotic. *BIRC7* expression, like other IAPs, is low in healthy and differentiated tissues, and high levels are found in the placenta, lymph nodes, spleen, embryonic tissues or tumors. Increased *BIRC7* was especially found in melanoma [32]. In our study, *BIRC7* was expressed only in 13 out of 42 human milk samples. More research is needed on the cause of the lack of expression of the *BIRC7* gene in some of the human milk stem cell samples tested.

In our research presented in this study, we have shown that the levels of *BIRC6* and *NAIP* gene expression in milk stem cells are significantly higher in women who gave birth before 37 weeks of pregnancy, compared to women who gave birth on time. No literature information was found assessing the effect of the term of pregnancy completion on the expression of genes from the IAP family in breast milk stem cells. IAP inhibitors of apoptosis have a wide range of functions. They can regulate and inhibit the apoptosis process. They participate in signal transduction, cell differentiation, and division, and also participate in the body’s immune response [14]. Studies have shown that the *BIRC6* mutation increases the risk of bacterial infections in children. Therefore, it can be concluded that the expression of the *BIRC6* gene in breast milk stem cells will affect the immunity of premature babies. The occurrence of the rs183868412 variant in combination with the increased expression of the *BIRC6* gene may determine a greater morbidity of prematurely born children and increase the risk of bacteremia [33]. We can speculate that the higher expression of the *BIRC6* and *NAIP* gene in milk stem cells in women who gave birth to premature babies results in a greater proliferation potential and a higher level of viability of these cells. However, this requires further studies, and cell viability was not assessed in this study. There is information in the literature that human milk stem cells can integrate with the child’s organism and differentiate, inter alia, into nerve cells, thanks to which they can contribute to the improvement of development and maintenance of homeostasis in a child, especially those born prematurely [25,34,35].

The results of our work proved a positive correlation between *XIAP* gene expression and *BIRC2* and *BIRC6.* The available scientific literature addresses the issue of Xiap gene expression, e.g., in the context of stem cells, in the treatment of brain damage in an animal model. *XIAP* overexpression has been shown to inhibit apoptosis of brain nerve cells and activate astrocytes [36]. In turn, *BIRC2* expression is critical for endothelial cell survival [37]. Taking these elements into account, it can be hypothesized that the correlation of expression of these genes in MSCs affects the development and regenerative processes of the most important systems of the newborn, namely, related to adaptive changes in postnatal life.

Stem cells derived from breast milk are an important component of human milk. Abd Allah et al. (2016) conducted research involving rabbits that were fed food with stem cells. It turned out that MSCs were not digested in the digestive tract and left it by diapedesis and then went to various organs, stayed there and stimulated organ development. The end result was a greater weight gain in the study group compared to the control group [38]. Ravera, S. et al. (2017) showed that UC-MSCs collected from premature infants showed a higher proliferative potential compared to full-term infants [39]. In our study, the average expression of *BIRC6* and *NAIP* genes in the milk stem cells of women who gave birth prematurely was statistically significantly higher than in women who gave birth at term. In in vivo and in vitro studies, neuronal apoptosis inhibitory protein (NAIP) turned out to be a strong modulator of cell survival [40]. According to the literature data, the number of stem cells in breast milk may vary depending on the gestational age. Changes in the composition of breast milk are intended to best support the development of the child [28]. On this basis, we can assume that the increased expression of *NAIP* in MSCs of breast milk born prematurely may be a response to the increased needs due to the immaturity of the neonate’s body. In women who experienced miscarriage, low *NAIP* expressions may suggest a lower cell survival capacity and a greater tendency to apoptosis. Isolation of MSCs from the breast milk of patients who experienced miscarriages could be associated with subsequent cell culture failures. In our work, we have shown that the properties of MSC in breast milk change with time since delivery. In colostrum and in milk isolated in the first week after parturition, MSC cells showed lower expression of *NAIP* than in the 4^th^ week after parturition or later. In the literature data, we also find information that the lactation phase and other factors (degree of satiety, breastfeeding, expressing milk, growth rate, infant’s needs, mother’s health, environment) cause differences in the composition of breast milk [24,25].

Such scientific reports further emphasize the importance of feeding premature babies with human milk. Scientists also note changes in the composition of milk in women who gave birth prematurely to those who gave birth at term. It has been suggested that this is to compensate for premature babies with an underdeveloped immune system. Interestingly, the composition of full-term and preterm milk becomes very similar over time [27]. It has been reported in the literature that preterm infants (born before 37 weeks of pregnancy) fed shortly after delivery with mother’s milk have a much lower percentage of necrotizing enterocolitis and infections. This suggests that the composition of milk at different stages of lactation is subject to cellular regulation, which corresponds to the current needs of the newborn [41]. Additionally, studies by Twigger et al. indicate that late gestational age (above 40 weeks) is correlated with higher expression of the α-LA and NESTIN genes, as well as lower expression of the *SOX2* pluripotency gene [41]. In our research, we also noticed differences in gene expression depending on the time of delivery; however, these conclusions concerned the expression of IAP family genes and not SOX2. Stem cells have tremendous potential for self-renewal and differentiation. When analyzing the conducted studies, a high level of pluripotency factors—an expression of the *POU5F1* and *SOX2* genes—are found. According to scientific reports, the expression level is related to the effect exerted by these factors. According to the work of Rizzino A et al., the expression level of the transcription factor Oct4, the product of the *POU5F1* gene, enables the enhancement of self-renewal at low levels, or the induction of differentiation at high values. Expression of proteins such as OCT4 or NANOG correlates with the cell remaining in an undifferentiated state [6,7]. Stem cells analyzed in this study showed high expression of *SOX2* and *OCT4*.

The research presents the results related to the regulation of stem cells by epigenetic mechanisms. Histone methylation or deacetylation are just some of them. Moreover, it was attempted to use this knowledge in oncological therapies by analyzing the ectopic expression of the *OCT4* and *NANOG* genes [42]. It can be speculated whether the presence of the expression of these genes in mother’s milk stem cells may be a reason to explore its protective anti-cancer effect. Nanog levels in embryonic stem cells (ESC) determine self-renewal (high level of expression) or susceptibility to differentiation signals (low level of expression) [6,43]. Despite the research on markers of pluripotency, it has not been established what causes these cells to remain undifferentiated [7].

The literature has shown a relationship between the expression of the *SOX2* gene in breast milk stem cells and clinical parameters related to the mother, such as the change in the size of the cup and the baby—the week of pregnancy in which delivery occurred. As a result of their research, Twigger et al. found a relationship between the expression of the *SOX2* gene and the week of pregnancy in which delivery occurred. The closer to the due date of delivery, the lower the expression of the *SOX2* gene was. They also described the relationship between *SOX2* gene expression and changes in breast size. The greater the change in cup size during pregnancy, the lower the expression of the *SOX2* gene [41].

Our previous studies have shown that the expression of the *SOX2* gene in Wharton’s jelly mesenchymal stem cells is significantly dependent on the age of the mother, the pH of the umbilical cord blood, the gestational week at which delivery occurred, and the birth weight of the newborn. We observed that the younger the woman, the lower the pH of the umbilical cord blood, the earlier the delivery took place and the lower the birth weight of the newborn, the higher the expression of the *SOX2* gene in MSCs [44,45]. However, this study did not show significant relationships of *SOX2* expression in milk stem cells with the analyzed clinical features.

*SOX2* and *OCT4* are involved in pluripotency and cell self-renewal. High levels of *SOX2* expression are also found in cells that have a high potential for differentiation and development [46]. *SOX2* expression decreases after cell differentiation. It can also be changed by various factors, such as *OCT4*, which can increase mir-21 and decrease the expression of this gene [47]. In our research, we showed a positive correlation between *SOX2* and *OCT4*, which would confirm the participation of these factors in cellular processes and would prove the pluripotent properties of MSCs in human milk.

In our previous work, we assessed the expression of *POU5F1* and *SOX2* genes in stem cells isolated from Wharton’s umbilical cord jelly. Our research was the first to show that the expression of the *POU5F1* gene in mesenchymal stem cells obtained from Wharton’s jelly of the umbilical cord is dependent on the age of the pregnant woman, the method of delivery and the use of oxytocin. Our previous studies show that the expression of *POU5F1* in mesenchymal stem cells decreases with each subsequent pregnancy and each subsequent birth. Wharton’s jelly stem cells collected from younger women and during the first delivery, as well as from patients treated with oxytocin, show a higher expression of *POU5F1* compared to subsequent deliveries, which can be considered to be characterized by a lower level of differentiation [48]. In this study, we observed that P*OU5F1* expression is statistically significantly higher in women delivering by caesarean section compared to vaginal delivery, which contradicts our previous observations. However, milk stem cells are harvested at a certain time after delivery and perhaps the effect of the physiological hypoxia that occurs during vaginal delivery is not as apparent, but this requires further research.

Studies by P. Singh et al. conducted on uterine tissues of adult mice show that MSC OCT4 (+) participate in the regeneration of the endometrium in physiological conditions and after mechanical trauma [49]. Presumably, this is due to the fact that a CC delivery is a major trauma to the uterine muscle compared to a vaginal delivery. Perhaps this is also related to the expression of *OCT4* in breast milk MSCs, which would explain our results.

Apart from the type of parturition, we did not show any significant relationship between *OCT4* expression in milk stem cells and clinical parameters.

In the studied milk stem cells, we noted a positive, very strong relationship between the expression of the examined pluripotency factors *SOX2* and *OCT4,* which is typical for stem cells with a high expression of pluripotency factors, and we also noted the occurrence of moderate and strong correlations between the expression of *SOX2* and *BIRC8, SOX2* and *XAF1, OCT4* and *BIRC7, OCT4* and *BIRC8, OCT4* and *XAF1*. The performed analyses suggest a relationship at the molecular level between selected IAPs and pluripotency factors, but this requires further, more detailed research.

There are attempts to exploit the potential of stem cell pluripotency in experimental therapies. An attempt has been made in the United States to use differentiated embryonic stem cells to form oligodendrocyte precursors that produce the myelin sheath [50]. In Kyoto, iPSC was used to experimentally treat macular degeneration with a satisfactory result [51]. This gives hope for an innovative and developmental approach to the clinical use of stem cells, also from human milk, as an alternative source of their acquisition.

Our research showed a statistically significantly higher expression of the *XAF1* gene in the stem cells of milk obtained from women who gave birth in a natural way compared to the material collected from women after caesarean section. In the case of the remaining genes from the IAP family, no dependence of expression on the mode of delivery was observed. No information has been found in the literature on the influence of the method of termination of pregnancy on the expression of IAP genes in milk stem cells. In the studies of Gil-Kulik et al., the relationship between the expression of selected IAPs in MSCs obtained from the umbilical cord and the type of delivery was observed. These studies showed a statistically significant relationship between the mode of delivery and the expression of *BIRC2* and *BIRC5* [14].

Performing the present study, a relationship between maternal age and the expression of the *XAF1* gene was observed. Its level has been shown to increase with age. The other tested genes showed no significant dependence of the level of expression with the age of the women from whom the milk was taken. Earlier studies by Gil-Kulik et al. on MSC obtained from the umbilical cord have shown that there is a negative correlation between *BIRC2*, *BIRC3* and *BIRC5* expression and maternal age [14].

Moreover, a statistically significantly higher expression of *BIRC2* and *BIRC6* in MSC from the milk obtained from women after the second pregnancy was demonstrated, a higher expression of *XAF1* in the material obtained from women after the first pregnancy, and a higher level of *BIRC6* and *NAIP* in women who gave birth prematurely.

Studies on MSC derived from the umbilical cord found no correlation between *BIRC2* and pregnancy sequence, while *BIRC6, XAF1*, and *NAIP* were not measured in this study [14].

All non-conformities may result from a different material from which the MSC was obtained for the above-mentioned studies. The differences in properties between the milk and the umbilical cord may affect the expression of IAP genes and, thus, the survival of these cells.

Additionally, our research proved the existence of a positive correlation between the expression of *BIRC2* and *BIRC3.* The confirmation of these results is the obtaining of the same relationship in MSC from the umbilical cord [14].

Liston et al. documented the expression of the *XIAP* gene in all cells of the human body, except for white blood cells. *XIAP* occurs in both mature and fetal tissues [52]. This is in line with our studies that showed *XIAP* expression in MSCs isolated from the breast milk of women after delivery.

Moreover, the expression of *BIRC5* in MSCs from milk has been demonstrated. This confirms previous studies in which the expression of this gene was detected in MSCs obtained from the umbilical cord. Moreover, in previous work, we speculated that *BIRC5* might be a new factor in stem cell pluripotency [23]. This study further shows that the stem cells express *BIRC5.*

The presented studies show the presence of higher levels of *BIRC6* and *XAF1* expression in MSCs obtained from women with a BMI higher than 30. The higher expression of *BIRC6* found in the milk stem cells of obese women may have a negative impact on the immunity of children in the same way as in the case of children born prematurely. In addition, *BIRC6* has been shown to negatively affect autophagy, which is likely to further weaken children’s immunity [53].

This may be due to the influence of obesity on stem cells. Bellows et al. observed an increase in the amount of MSC in the case of people with BMI above normal, which may lead to increased expression of *BIRC6* and *XAF1* [54]. In our study, we took into account maternal BMI, and it was shown that obese women expressed higher *BIRC6* and *XAF1*, while women with normal weight showed higher expression of the *BIRC5* gene. This may be due to the difference in the composition of the milk of these women. Some studies show that infants of obese mothers are exposed to higher levels of leptin, insulin, TNF-α, IL-6 and adenine compared to lean women. This could also explain the weight-dependent changes in stem cell composition [55,56]. Studies by Y. Nishimura et al. in which the effect of mouse diet on the expression of Xaf1 in mouse pancreatic islets was examined. They showed an increase in expression in animals consuming a high-fat diet, whose weight increased, compared to low expression of this gene in mice with a normal diet and lower weight. Although these studies have not been conducted in humans, it can be assumed that excess body weight is associated with an increase in *XAF1* expression not only in pancreatic islet cells, but also in breast milk stem cells, and possibly in other body cells. In the future, this may be related to the increase in the development of type 2 diabetes, associated with apoptosis of pancreatic β islets [57].

The effect of MSCs on cytostatic-induced AKI has been studied, showing beneficial effects associated with the re-entry into the cell cycle of previously damaged renal tubular cells, the authors point to the involvement of overexpression of anti-apoptotic genes such as *BIRC8* [58]. According to the results of our research, increased expression of XAF1 is observed in women who gave birth vaginally, which also positively correlates with the patient’s age, so it can be associated with a higher risk of hypoxia and, thus, stress for the cells. XAF1 functions as a co-activator of stress-triggered transcription factors and enhances cellular sensitivity to apoptotic stresses through a p53-independent mechanism [59]. Based on these data, it can be concluded that the positive correlation of *XAF1* and *BIRC8* gene expression plays a protective role, guaranteeing the simultaneous processes of repairing damaged cellular elements and destruction of cells unable to self-repair, which may support the child’s adaptation to the external environment.

Hassiotou and Hartmann [24] discovered the presence of CD34+ hematopoietic stem/progenitor cells from the mothers bloodstream that was demonstrated in colostrum and breast milk.

A statistically significantly higher expression of *BIRC5* in colostrum-derived cells compared to cells obtained from transitional and mature milk was demonstrated. It can be assumed that it is related to cell maturation. Previous studies have shown a decline in *BIRC5* expression with the degree of cell maturity [23]. This would explain why colostrum, as a product preceding milk production, is characterized by a higher expression of this gene.

Increased expression of *XAF1* was observed in MSCs obtained from women after vaginal delivery. Perhaps it is related to the hypoxia that develops as a result of natural childbirth. This is confirmed by the studies by Russel et al., in which an increase in the level of *XAF1* expression in neurons after hypoxia caused by ischemia was observed [60].

## 4. Materials and Methods

### 4.1. Characteristics of the Study Group

The research was carried out on samples of human milk collected from 42 patients hospitalized at The Clinic of Obstetrics and Perinatology of the Independent Public Clinical Hospital No. 4 in Lublin.

Samples of milk were collected in the morning in the amount of 5 mL in the period from the 2nd to the 136th day after delivery, the average time of sampling was 9.5 days after delivery. The samples were divided into two groups depending on the time that has elapsed since delivery: colostrum—21 samples in the period from day 2 to 5 after delivery; in the case of milk—21 samples in the period from day 7 to day 136 after delivery.

The study groups are independent. Only healthy women were included in the study. The average age of the women was approximately 30 years.

The characteristics of the study group are presented in Table 2, Table 3, Table 4, Table 5, Table 6, Table 7 and Table 8.

The tests were carried out with the consent of the Local Bioethical Commission No. KE-0254/128/2014.

### 4.2. Methods

#### 4.2.1. Isolation of Stem Cells

The research was carried out in stem cells derived from mother’s milk, which were isolated using the method proposed by Hassiotou [61].

Stem cells were isolated from the milk by centrifugation; each sample was diluted 1: 1 with a buffered saline solution (PBS) without Ca and Mg ions (Biomed Lublin, Poland), and then centrifuged for 20 min, at 20 °C, and 805 g. After centrifugation, the fat layer and the supernatant were discarded, and the cell pellet was washed three times with PBS solution [61].

#### 4.2.2. Cell Culture and Flow Cytometry

Cells were cultured under adherent conditions (Table 9) and the fibroblast-like shape of the cells was demonstrated (Figure 2) and the ability of cells to adhere to the plastic walls.

#### 4.2.3. Immunophenotyping of Mesenchymal Stem Cells from Milk

The stem character of the isolated cells was confirmed. The cytometric test showed the presence of CD73, CD90, CD105 and CD146 surface antigens (Figure 1a–d) characteristic of mesenchymal stem cells (cytometric analysis was carried out following protocol presented in the paper by Walecka et al. [62]. High expression of the *POU5F1* and *SOX2* genes was demonstrated in the cells tested, which are the main factors of stem cell pluripotency.

A 100 μL suspension of milk mesenchymal stem cells obtained from in vitro culture was added to the DURAClone SC Mesenchymal Tube (C34369, BeckmanCoulter, Bangalore, Karnataka, India) and vortexed for 6 s. Cells were incubated in the dark for 15 min, at room temperature. DURA Clone MSC tubes contain lyophilisate of a panel of 9 monoclonal antibodies (CD90-FITC, CD73-PE, CD34-ECD, CD146-PC5.5, CD105-PC7, CD45-APC-AF750, CD31-Pacific Blue, CD14-Krome Orange, CD19-Krome Orange) dedicated to the determination of characteristic MSC antigens. The test is based on the binding of monoclonal antibodies to specific antigenic determinants of MSC. After incubation, 2 mL of buffered saline (PBS) without calcium and magnesium ions were added. It was then centrifuged at 200× *g* for 5 min. The supernatant was discarded, and the cell pellet was dissolved in 400 µL of buffered saline (PBS) without calcium and magnesium ions. The labeled cells were placed on the carousel of a Navios flow cytometer (BeckmanCoulter) and subjected to cytometric analysis.

The viability of the stem cells was also assessed cytometrically using propidium iodide (Figure 1e).

#### 4.2.4. Molecular Analysis of the Expression of Genes from the IAP Family

Total cellular RNA was isolated by the modified method of Chomczyński [63] from the obtained cells using TRI Reagent (Sigma), isopropanol (Sigma), chloroform (Sigma), ethyl alcohol (Poch, Poland). The RNA extract was spectrophotometrically evaluated.

The reverse transcription reaction was carried out as recommended by the manufacturer, using the High-Capacity reagent cDNA Transcription Kits with RNase Inhibitor (Applied Biosystems, USA) and 1 μg of isolated RNA.

Relative gene expression levels of *NAIP, BIRC2, BIRC3, BIRC5, BIRC6, BIRC7, BIRC8, XIAP*, *XAF1*, *OCT4* and *SOX2* were examined by the qPCR method using commercially available TaqMan probes (Applied Biosystems, USA): *GAPDH*: Hs99999905_m1; for *NAIP* gene: Hs03037952_m1; for *BIRC2* gene: Hs00357350_m1; for *BIRC3* test gene: Hs00154109_m1; for *BIRC5*: Hs00153353_m1; for *BIRC6:* Hs00212288_m1; for *BIRC7:* Hs01086675_m1; for *BIRC8*: Hs01057786_s1; for *XIAP:* Hs00236913_m1; for *XAF1*: Hs00213882_m1; for *OCT4:* Hs00242302_m1; and for *SOX2* gene: Hs00193931_m1. *GAPDH* was an endogenous control gene. The expression of the examined genes was calculated from the formula RQ = 2^−ΔΔCT^ [64]. The Expression Suite Software 1.0.3 was used to calculate gene expression level (Life Technologies). Detailed procedures for the RNA isolation and qPCR reaction shave have been described in our previous work [23,48].

#### 4.2.5. Statistical Analysis

Statistical analysis was performed using STATISTICA 13 software. Due to the normal distribution of the examined variables, Student’s *t*-test was used to calculate the differences in the gene expression in the tested groups, and Pearson’s correlation test was used to calculate correlations.

## 5. Conclusions

Our research has shown that clinical data collected in medical history, such as maternal BMI, maternal age, number of pregnancies and deliveries, miscarriages, way of delivery, time of delivery and time of milk collection for research, have an impact on changes in the expression of genes from the IAPs family. It has been proven that with increasing maternal age, the expression of the *XAF1* gene increases in obese women during their first pregnancy and those who gave birth by force of nature. Higher expression of the *BIRC6* gene was found in women who were pregnant again, and when the delivery took place before 37 weeks of pregnancy. Research on the expression of the *BIRC7* gene, which was expressed only in 13 out of 42 samples, should be expanded. Additionally, this work demonstrated positive relationships between the gene expression of *BIRC7, BIRC8* and *XAF1* and the main factors of pluripotency, which require further explanation.

It should be emphasized that stem cell research is still incomplete. Our work is the first to focus on the expression of apoptosis inhibitory genes in mother cells derived from breast milk; however, these are preliminary studies that will continue.

## Figures and Tables

**Table 1:** Analysis of the mutual correlations between the expression of the tested genes and the expression of the tested genes and parameters such as maternal age, maternal BMI, number of pregnancies and deliveries, gestation week, sampling time, and newborn bodyweight. * *p* < 0.05.

Parameter	logRQ *BIRC2*	logRQ *BIRC3*	logRQ *BIRC5*	logRQ *BIRC6*	logRQ *BIRC7*	logRQ *BIRC8*	logRQ *NAIP*	logRQ *XAF1*	logRQ *XIAP*	logRQ OCT4	logRQ SOX2
logRQ *BIRC2*	1.000	0.449 *	0.175	0.819 *	0.338	−0.056	0.650 *	−0.087	0.563 *	−0.112	−0.222
logRQ *BIRC3*	0.449 *	1.000	0.120	0.189	0.402	0.362 *	0.426 *	0.488 *	0.308	0.134	0.134
logRQ *BIRC5*	0.175	0.120	1.000	0.396 *	0.648 *	0.319	0.101	0.034	0.165	0.189	0.193
logRQ *BIRC6*	0.819 *	0.189	0.396 *	1.000	0.272	−0.228	0.577 *	−0.308	0.543 *	−0.268	−0.314
logRQ *BIRC7*	0.338	0.402	0.648 *	0.272	1.000	0.488	0.324	0.479	0.456	0.591 *	0.380
logRQ *BIRC8*	−0.056	0.362 *	0.319	−0.228	0.488	1.000	−0.108	0.484 *	−0.149	0.821 *	0.798 *
logRQ *NAIP*	0.650 *	0.426 *	0.101	0.577 *	0.324	−0.108	1.000	−0.051	0.314	−0.182	−0.301
logRQ *XAF1*	−0.087	0.488 *	0.034	−0.308	0.479	0.484 *	−0.051	1.000	−0.160	0.568 *	0.492 *
* logRQ *XIAP*	0.563 *	0.308	0.165	0.543 *	0.456	−0.149	0.314	−0.160	1.000	−0.251	−0.194
logRQ OCT4	−0.112	0.134	0.189	−0.268	0.591 *	0.821 *	−0.182	0.568 *	−0.251	1.000	0.962 *
logRQ SOX2	−0.222	0.134	0.193	−0.314	0.380	0.798 *	−0.301	0.492 *	−0.194	0.962 *	1.000
Mother’s age	−0.133	0.113	0.093	−0.106	0.442	0.113	−0.121	0.438 *	−0.002	0.114	−0.039
BMI	−0.265	0.100	−0.202	−0.587 *	0.143	−0.113	−0.122	0.667	−0.323	−0.052	−0.239
The day after the birth	0.306	0.118	−0.159	0.285	−0.332	−0.051	0.306	−0.494 *	−0.094	−0.064	−0.088

**Table 2:** Parameters describing the study group.

Parameter	N	Mean	Median	Min	Max	SD	SE
Mother’s age (year)	42	30.15	29.50	22.00	42.00	4.67	0.69
BMI		27.96	26.60	23.90	37.50	4.05	0.91
The day after the birth		20.50	9.50	2.00	136.00	30.53	4.15
Number of pregnancies		1.93	2.00	1.00	4.00	0.78	0.12
Number of deliveries		1.71	2.00	1.00	3.00	0.77	0.12
Week of pregnancy		31.91	32.50	24.00	40.00	5.34	0.80
Newborn weight (g)		2106.86	1880.00	480.00	4550.00	1127.89	157.94

**Table 3:** Number of pregnancies.

Number of Pregnancies	Number of Patients	%
1	13	31
2	20	48
3	8	19
4	1	2

**Table 4:** Number of deliveries.

Number of Deliveries	Number of Patients	%
1	20	48
2	14	33
3	8	19

**Table 5:** Miscarriages.

Miscarriages	Number of Patients	%
yes	9	21
no	33	79

**Table 6:** Way of delivery.

Way of Delivery	Number of Patienst	%
CC	35	83
ND	7	17

**Table 7:** Prematurity.

Prematurity	Number of Cases	%
yes	27	64
no	15	36

**Table 8:** The sex of the child.

The Sex of the Child	Number of Cases	%
F	21	50
M	21	50

**Table 9:** Conditions for the cell culture of stem cells isolated from mother’s milk.

Source of Cells	Breas Milk (after Isolation by Centrifugation)
Type of cell culture	Adherent (medium volume—10 mL)
Culture vessel	Culture bottle; surface area of the bottom: 25 cm^2^; TC Flask T25, Cell+; Sarsted; Germany
Culture medium	DMEM (1×) + GlutaMAX™-I [+] 1 g/L D-Glucos [+]; Pyruvate Gibco; UK
Serum	Heat Inactivated FBS; Gibco; USA
Antibiotics	Amphotericin B 250 μg/mL + Penicillin/Streptomycin (100×); PAA; Austria
Temperature	37 °C
O2 concentration	15%
CO_2_ concentration	5%
Moisture	95%

## Data Availability

The data used to support the findings of this study are included in the article.
